# Supplementary figures and images for: Stage-specific MCM protein expression in Trypanosoma cruzi: insights into metacyclogenesis and G1 arrested epimastigotes
Source: Front Cell Infect Microbiol. 2025 May 26;15:1584812. doi: 10.3389/fcimb.2025.1584812 (PMC12146363; doi:10.3389/fcimb.2025.1584812)

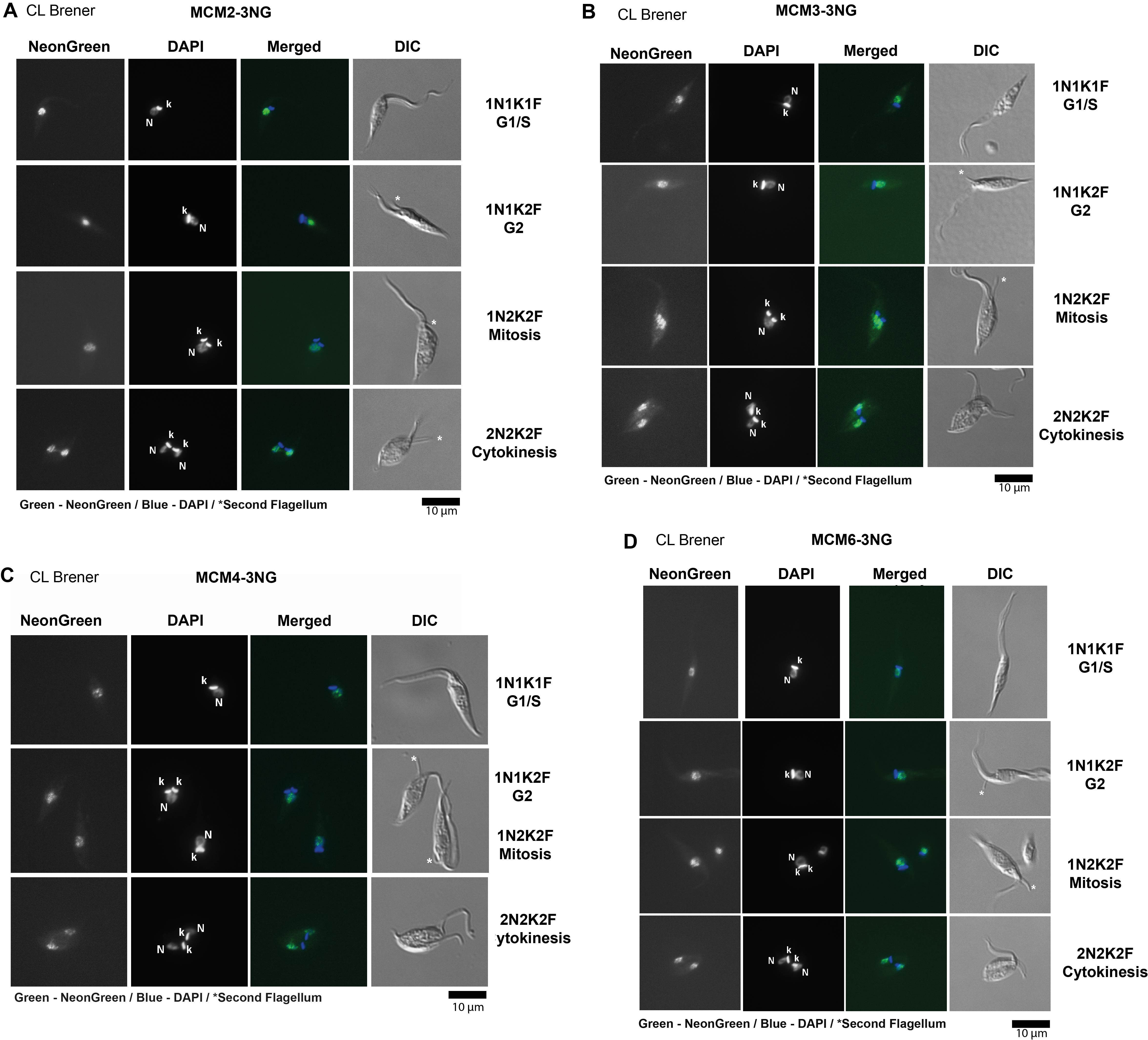

Supplement: Supplementary Figure 1 — Fluorescence imaging of T. cruzi CL Brener strain epimastigotes modified using CRISPR/Cas9. T. cruzi epimastigotes were genetically modified by CRISPR/Cas9 to incorporate three copies of the mNeonGreen gene at the 3’ end of the MCM2, MCM3, MCM4, and MCM6 genes. Fluorescence microscopy was employed to capture images of mNeonGreen fluorescence (green), DAPI-stained DNA (blue), and Differential Interference Contrast (DIC). Cells at different stages of the cell cycle are shown. The black scale bar represents 10 µm. N indicates the nucleus, K indicates the kinetoplast, and F indicates the flagellum. (A) MCM2-3mNG cell line; (B) MCM3-3mNG cell line; (C) MCM4-3mNG cell line; (D) MCM6-3mNG cell line. [file Image1.jpeg]

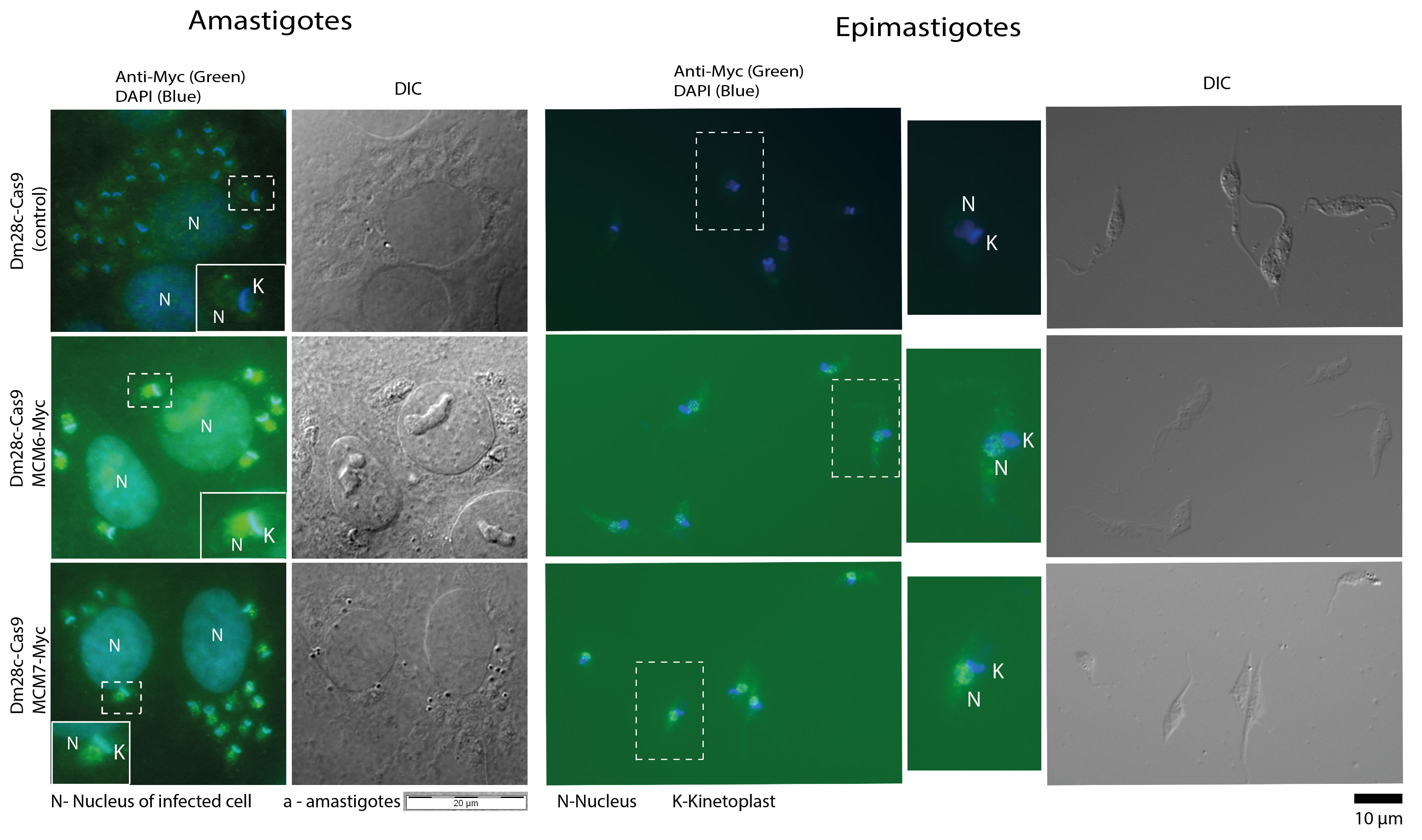

Supplement: Supplementary Figure 2 — Immunofluorescence imaging of T. cruzi Dm28c strain epimastigotes and amastigotes genetically modified using CRISPR/Cas9. The epimastigotes were modified by inserting three copies of the Myc sequence at the 3’ end of the MCM6 and MCM7 genes. Immunofluorescence microscopy was performed to visualize anti-Myc staining (green), DAPI-stained DNA (blue), and Differential Interference Contrast (DIC) images. Here are shown raw images of intracellular amastigotes and epimastigotes from: Dm28c-Cas9 (control), Dm28c-MCM6-Myc, and Dm28c-MCM7-Myc cell lines. The black scale bar represents 10 µm, and the white scale bar represents 20 µm. Dashed rectangles highlight cells that are 2x magnified. For amastigotes, magnified images are on the bottom right (control and MCM6-Myc) and bottom left (MCM7-Myc). For epimastigotes, the magnified cells are located to the left of the immunofluorescence image. N denotes the nucleus and K the kinetoplast. [file Image2.jpeg]

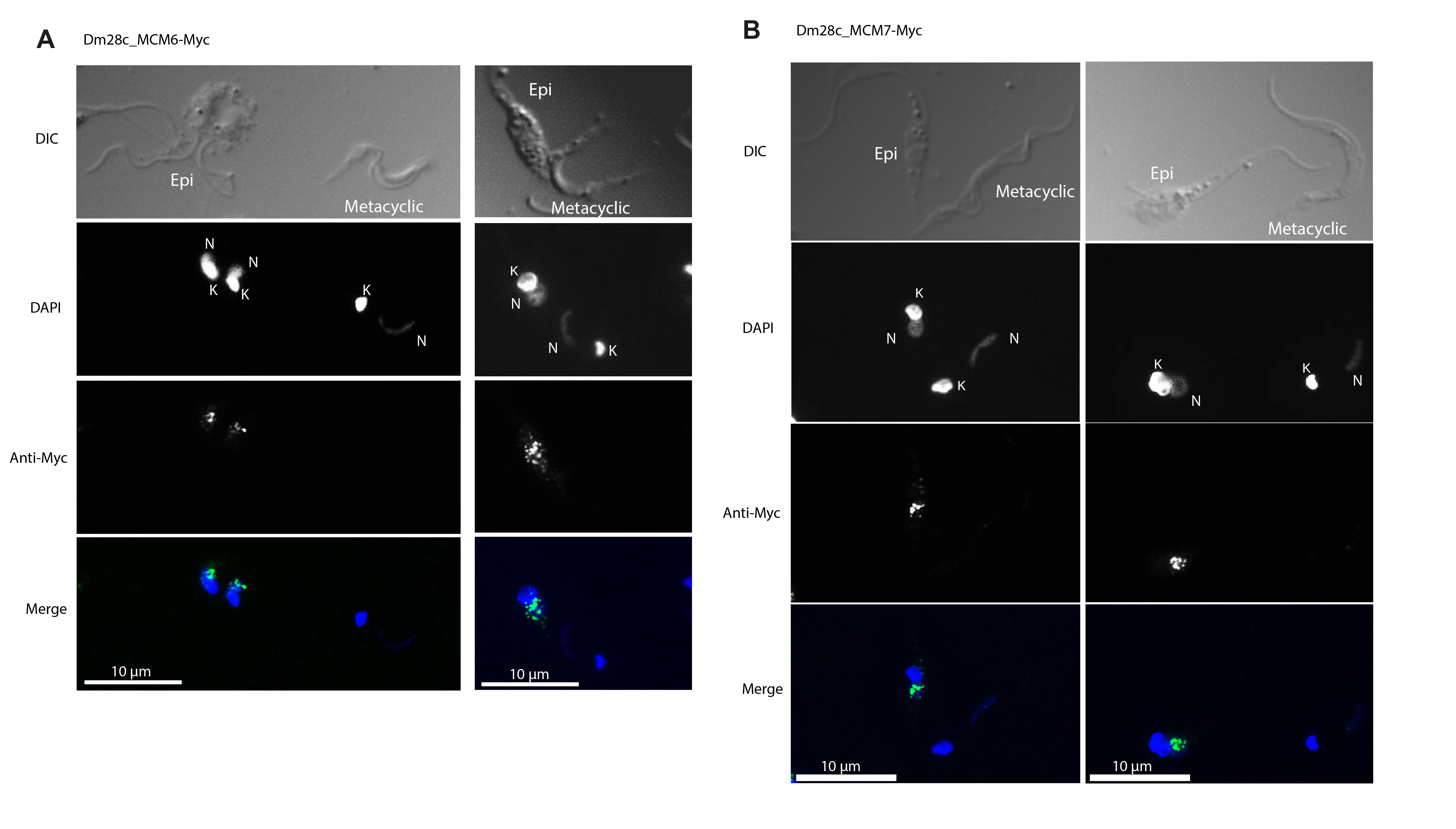

Supplement: Supplementary Figure 3 — Immunofluorescence imaging of T. cruzi Dm28c cells after metacyclogenesis. Epimastigotes from the MCM6-Myc and MCM7-Myc cell lines (Dm28c strain) were subjected to metacyclogenesis. Immunofluorescence staining was performed using an anti-Myc antibody. The images show both differentiated metacyclic trypomastigotes and non-differentiated epimastigotes. (A) MCM6-Myc cell line and (B) MCM7-Myc cell line. Anti-Myc staining is shown in green, DAPI-stained DNA in blue, and Differential Interference Contrast (DIC) images are included. The white scale bar represents 10 µm. N denotes the nucleus, K the kinetoplast. [file Image3.jpeg]
